# The Role of Extracellular Vesicles as Allies of HIV, HCV and SARS Viruses

**DOI:** 10.3390/v12050571

**Published:** 2020-05-22

**Authors:** Flavia Giannessi, Alessandra Aiello, Francesca Franchi, Zulema Antonia Percario, Elisabetta Affabris

**Affiliations:** Department of Science, Roma Tre University, 00146 Rome, Italy; flavia.giannessi@uniroma3.it (F.G.); alessandra.aiello@uniroma3.it (A.A.); fra.franchi@stud.uniroma3.it (F.F.); zulema.percario@uniroma3.it (Z.A.P.)

**Keywords:** extracellular vesicles, exosomes, HIV, HCV, SARS viruses, coronaviruses

## Abstract

Extracellular vesicles (EVs) are lipid bilayer-enclosed entities containing proteins and nucleic acids that mediate intercellular communication, in both physiological and pathological conditions. EVs resemble enveloped viruses in both structural and functional aspects. In full analogy with viral biogenesis, some of these vesicles are generated inside cells and, once released into the extracellular milieu, are called “exosomes”. Others bud from the plasma membrane and are generally referred to as “microvesicles”. In this review, we will discuss the state of the art of the current studies on the relationship between EVs and viruses and their involvement in three important viral infections caused by HIV, HCV and Severe Acute Respiratory Syndrome (SARS) viruses. HIV and HCV are two well-known pathogens that hijack EVs content and release to create a suitable environment for viral infection. SARS viruses are a new entry in the world of EVs studies, but are equally important in this historical framework. A thorough knowledge of the involvement of the EVs in viral infections could be helpful for the development of new therapeutic strategies to counteract different pathogens.

## 1. Introduction

Extracellular vesicles (EVs) are heterogeneous and polyhedral lipid bilayer-enclosed structures released by healthy, malignant or infected cells into the extracellular environment, with different origins, sizes and compositions [1]. Soon after their first observation by Chargaff and West in 1940s, EVs were considered as mere cellular “dust”. Gradually, numerous studies have recognized them as multi-molecular messengers acting in both autocrine and paracrine manners, and even at great distance, modifying the activity and/or phenotype of recipient cells [2]. EVs have been isolated from most cell types and biological fluids including blood, urine, saliva, breast milk, and cerebrospinal and synovial fluid [3,4,5,6,7]. In physiological conditions, they are involved in antigen presentation [8], neuronal communication and protection [9], blood coagulation [10], wound healing [11], sperm maturation [12] and regulation of the immune response against the fetus during pregnancy [13]. On the other hand, EVs play a key role also in pathogenic processes including cancer [14], autoimmune diseases [15], inflammation [16], as well as in viral infections [17]. Pathogens like viruses seem to take advantage of the natural inclination of host cells to release EVs to transport their own genetic material or proteins, thus avoiding their recognition as non-self-molecules by the immune system. Therefore, EVs can play significant roles during viral infections by promoting the survival and propagation of the virus inside the host.

In this review, we provide an overview of the relationship between EVs and enveloped viruses, focusing our attention on HIV, HCV and SARS viruses.

## 2. Definition, Biogenesis and Composition of EVs

Based on their biogenesis, EVs have been divided into three subgroups: exosomes, microvesicles and apoptotic bodies. Exosomes correspond to intraluminal vesicles (ILVs), which are generated in late endosomes by the inward invagination of their membranes, giving rise to the formation of high-density structures named multivesicular bodies (MVBs). Once formed, ILVs can meet two different fates: they can be degraded when MVBs fuse with lysosomes, or they can be released into the extracellular space upon fusion of MVBs with the plasma membrane. In the latter case, ILVs are called exosomes (size between 30–150 nm) (reviewed by [18,19]). The term microvesicles (size between 50–1000 nm) generally refers to vesicles that bud directly from the plasma membrane, while apoptotic bodies (size between 50 nm to 5 μm) are vesicles generated by cells undergoing apoptosis [19,20].

Among the different types of EVs, exosomes are the best characterized. However, the processes leading to the generation of ILVs in MVBs and their fusion with the plasma membrane are not completely known. To date, two independent pathways have been proposed. The first one is accomplished by components of the Endosomal Sorting Complex Required for Transport (ESCRT), a molecular machinery made up by four multiprotein complexes (ESCRT-0, -I, -II, -III) and accessory proteins (i.e., Alix and VPS4) [21,22]. The second pathway for the biogenesis of exosomes is ESCRT-independent and involves tetraspanins (such as CD9, CD63, CD81 and CD82), a superfamily of proteins characterized by four transmembrane domains, and lipid molecules, such as ceramide, a conic lipid that facilitates membrane invagination [23,24]. The biogenesis of microvesicles differs considerably from that of exosomes. Prior to their shedding at the plasma membrane, cytoplasmic protrusions are generated by the cell, which undergoes fission events and, finally, microvesicles pinch off the cellular membrane [25]. The mechanisms underlying these shedding events are not well elucidated yet; however, microdomain-induced budding processes seem to be involved in their secretion. Recently, Shurer and colleagues suggested also a role for glycocalyx in regulating curved membrane features and driving the secretion of EVs ranging in size from approximately 100 nm to 400 nm [26]. Not by chance, enterocytes, reactive astrocytes, dendritic cells, and tumor cells, on whose surfaces mucins and hyaluronan polymers are densely arrayed, usually secrete high levels of vesicles [27,28,29,30,31,32]. According to what was reported by Shurer et al. [26], the glycocalyx would enable cytoskeletal filaments to extend and stabilize thin protrusions from the plasma membrane, and then spontaneous curvature imposed by the glycocalyx would induce the formation of membrane pearls that spontaneously fissure to release vesicles.

The important role played by EVs as potent vehicles of intercellular communication is mainly linked to their ability to carry a wide range of biological macromolecules such as proteins, lipids, and nucleic acids. Regarding nucleic acids, DNA fragments, single and double-stranded DNAs, mitochondrial DNA and RNA species, such as mRNAs, miRNAs and a great variety of small non-coding RNAs, have been detected in EVs [33,34,35]. Notably, emerging studies have also identified the release of EVs as a potential mechanism by which cytokines/chemokines can be secreted. Representative examples are Interleukins 1β (IL-1β) and IL-18, both secreted upon inflammasome activation, macrophage migration inhibitory factor (MIF), IL-32 and Tumor Necrosis Factor (TNF) family members. Interestingly, Interferon family members (IFNs) have also been detected in EVs (for a comprehensive review, see [36]).

Interestingly, in addition to self-molecules, EVs can be carriers of microbial components, including viral ones [34]. The encapsulation of molecules, both self and non-self, into EVs could protect them from enzymatic degradation and the recognition as danger signals during their transit into the extracellular milieu, thus facilitating their delivery at distant target cells.

## 3. EVs and Viruses: Close Relatives?

In recent decades, the similarity between EVs and viral particles has become increasingly evident. Viruses and EVs share different aspects such as size, structural and biochemical composition, and the transport of bioactive molecules within cells [34,35]. Like EVs, viruses present a size ranging from 30 to 1000 nm, starting from the small ones, such as poliovirus and hepatitis A virus (HAV) particles, which possess a diameter of about 30 nm, all the way to hepatitis C virus (HCV) of about 50 nm, and HIV or SARS viruses that are about 100–120 nm. Finally, mimiviruses have a size of about 400 nm. Furthermore, EVs and some viruses have morphological similarities: as previously described, EVs are double-membrane-enclosed entities and enveloped viruses are also surrounded by a lipid membrane acquired from the cell. Interestingly, they possess a similar lipid composition enriched in glycosphingolipids and cholesterol, as well as a similar protein content. Notably, both EVs and viruses carry nucleic acids; while viruses present single or double-stranded RNA or DNA genomes, which are carried and protected inside their capsid, EVs can transport a variety of nucleic acids [35,37,38]. EVs and enveloped viruses also share similar biogenesis processes since both are generated in the endosomal network or bud from the plasma membrane using specific pathways [18]. For example, some retroviruses such as HIV hijack the cellular vesiculation machinery to favor their own replication and budding. In this regard, it has been reported that the endosomal sorting complex (ESCRT), the same that mediates the inward invagination of ILVs in MVBs, is also involved in the budding and release of HIV particles [39,40]. Moreover, just as EVs can be generated from ESCRT-independent pathways, some viruses bud from specific membrane domains [41]. These domains, called lipid rafts, are enriched in glycosphingolipids, cholesterol and ceramide. In addition, proteins like tetraspanins are stored in these domains and form clusters among themselves and other transmembrane and cytosolic proteins, thus inducing inward budding of the microdomains in which they are enriched [42]. As previously mentioned, specific glycocalyx compositions also play a role in vesicle release; however, glycocalyx can be also involved in other membrane processes, including the absorption of some viruses [43]. In this regard, some viruses have evolved to exploit specific glycans to enter cells, like human rotaviruses that bind the blood group A antigens [44]. Instead, in the case of HIV [45], Ebola virus [46], HCV [47], as well as influenza [48] or Severe Acute Respiratory Syndrome (SARS) viruses [49], the viruses themselves present glycans on their surface. Their presence on viral surfaces is exploited by immune cells, such as macrophages or dendritic cells, to phagocyte virions. In turn, Ebola [46] and SARS viruses [49] take advantage of this anti-viral system to enter and replicate in macrophages and dendritic cells. On the other hand, glycans are also used by viruses to create a shield that hides viral epitopes to immune cells, as happens with HIV, known to have the highest density of glycans attached to its surface proteins [50], and the Lassa virus [51].

The substantial overlap of the biogenesis processes provides a plausible explanation for the similar composition observed between EVs and enveloped viruses [39]. Furthermore, both EVs and enveloped viruses can bind to the plasma membrane of recipient cells and, after fusion events, directly with the surface membrane or after endocytosis, they release their luminal cargo into the cytosol, influencing cell activity [18]. In this respect, in a similar manner to the viral envelope proteins, EV surface proteins, such as the intercellular adhesion molecule 1 (ICAM-1), mediate the adhesion and internalization of EVs in target cells [52]. Therefore, both EVs and viruses can be considered as bioactive structures able to influence the cellular behavior.

The presence of multiple similarities between viruses (in particular retroviruses) and EVs, immediately triggered conjecture on the real relationship between vesicles and viruses. For this reason, two alternative theories have been proposed. The first one, called the “Trojan exosome hypothesis”, states that retroviruses are vesicles evolved following a mutation of the *gag* gene, which was originally encoded by an integrated retro-transposon that directed its expression product towards the route of vesicle generation. In this perspective, the typical characteristics of retroviruses would have been acquired by evolutionary divergence; the pre-existing biogenesis mechanism of vesicle production would have been used to form viral particles [53]. The second theory does not associate viruses to modified exosomes. It justifies the similarities, giving more importance to the phenomenon of convergent evolution, which would lead to the sharing of the same biogenesis pathways for vesicles and viruses [54]. Both theories provide a plausible justification for the affinities observed between viruses and EVs. However, regardless of their possible origin, these affinities certainly have a negative impact on immunological surveillance in the host, since viruses, during infections, can take advantage of these affinities for escaping the immune system by mimicking vesicle composition and behavior [55].

The remarkable resemblance between EVs and viruses has caused quite a few problems in the studies focused on the analysis of EVs released during viral infections. Nowadays, it is an almost impossible mission to separate EVs and viruses by means of canonical vesicle isolation methods, such as differential ultracentrifugation, because they are frequently co-pelleted due to their similar dimension [56,57]. To overcome this problem, different studies have proposed the separation of EVs from virus particles by exploiting their different migration velocity in a density gradient or using the presence of specific markers that distinguish viruses from EVs [56,58,59]. However, to date, a reliable method that can actually guarantee a complete separation does not exist.

## 4. Vesicles as Mediators of a Suitable Environment for Viral Infections

**Figure 1:**
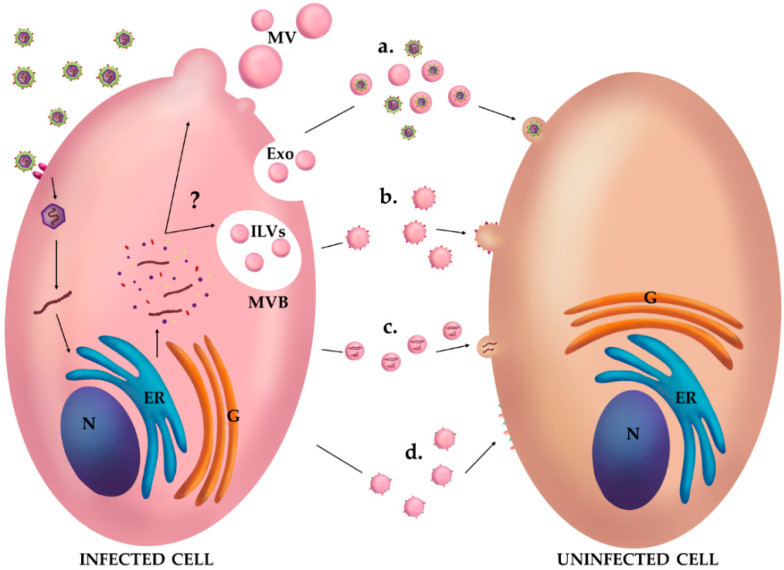
EVs are vehicles for the communication between infected and uninfected cells. During viral infections, virus enters cells and exploits the vesicular biogenesis machinery to release EVs, microvesicles (MV) and exosomes (Exo) with a modified composition to favor its own pathogenesis. EVs can carry (**a**) entire viral particles; (**b**) different viral proteins, such as the envelope ones; (**c**) nucleic acids including viral genomes, microRNAs and small non-coding RNAs and (**d**) host cell proteins, whose production is induced by the virus. Finally, EVs are internalized through different mechanisms and their luminal content released into the cytosol of the recipient cells. Nucleus (N); endoplasmic reticulum (ER); Golgi complex (G); multivesicular bodies (MVB); intraluminal vesicles (ILVs).

Studies conducted on exosomes and other EVs, isolated during a variety of infections caused by bacteria, parasites and viruses, have evidenced changes in the composition and biological activity of EVs [34]. In recent years, the relevance of vesicles in viral infections has been strongly highlighted, because EVs may incorporate viral proteins and/or fragments of viral RNAs, carrying them from infected cells to target ones [23,33,60]. Importantly, even if the viral hijacking of EVs contributes to create a suitable environment for viral survival through the suppression and evasion of the immune response, EVs can be involved in the induction of an antiviral response. Therefore, vesicles can play a dual role—both supporting viral spreading and inducing immunological protection [34]. Next we focused our attention on how vesicles can support viruses during infections. Some picornaviruses, such as HAV, Coxsackie B virus and Enterovirus 71 (EV71), can be released inside vesicles [61,62,63,64,65] (see Figure 1a). They are non-enveloped viruses but, when released inside EVs, they acquire a kind of “cellular envelope”. EV enveloped viruses probably take advantage of the membrane coating to avoid the recognition by neutralizing antibodies. In addition, these viruses could use cellular surface proteins to extend their own tropism, thus succeeding in reaching the most disparate districts in the host [33]. Instead, HIV and HCV seem to exploit EVs both directly and indirectly. They directly manipulate the machinery of vesicular biogenesis to enhance viral replication. Indirectly, they can charge exosomes and other vesicles with different viral components, thus favoring viral pathogenesis [23,66] (see Figure 1b,c). The dynamics of the influence of EVs on HIV and HCV infection will be discussed later and in detail.

Another well-known example is Epstein–Barr virus (EBV), a DNA virus that exploits vesicular production to block the antiviral response. As happens in HIV and HCV infections, EBV-infected cells release vesicles enriched with viral proteins, including Latent Membrane Protein 1 (LMP1), a pro-oncogenic protein that acts as deregulator of cellular transduction pathways by promoting EBV-infected B lymphocyte transformation and immortalization, as well as a global immune modulation [33,67,68,69]. LMP1 was found in vesicles collected from in vitro infected cells and from serum of patients with EBV-associated nasopharyngeal carcinoma [67,68,70]. A common belief is that LMP1 is selectively charged into EVs thanks to its localization in lipid rafts and its interaction with CD63, a well-known tetraspanin abundantly found in vesicles [71,72,73]. LMP1-containing EVs secreted by B cells inhibit T and natural killer (NK) cell proliferation, thus reducing the immune response against the virus [68,74]. Also, these EVs upregulate the expression of adhesion molecules in uninfected cells, increasing their susceptibility to the infection [75]. Moreover, EVs released from EBV-infected cells can contain viral nucleic acids [76,77]. In particular, different RNAs have been found: miR-BART15-3p, which induces apoptosis in target cells, including the immune ones [78]; miRNA BHRF1, which suppresses the expression of the chemokine CXCL11 involved in antiviral activity [79]; the non-coding RNAs, EBER1 and EBER2, that support the survival and carcinogenesis of infected cells by avoiding cell apoptosis [80].

Some viruses do not influence the encapsulation of viral proteins into vesicles, but control the packaging of host factors (see Figure 1d). This occurs in the case of Kaposi’s sarcoma-associated herpesvirus (KSHV/HHV-8). EVs released by KSHV-infected cells are enriched in metabolic proteins, such as lactate dehydrogenase, and in proteins affecting the immune system such as the cleaved forms of IL-1 and IFI16 [81,82]. Consequently, KSHV-EVs alter the metabolism and the innate immune response in recipient cells, facilitating viral persistence.

Cytomegalovirus (CMV) and herpes simplex virus 1 (HSV-1) usually exploit modified EVs as well. For instance, EVs from CMV-infected cells deliver proteins, such as lectin and dendritic cell-specific intercellular adhesion molecule-3 grabbing non-integrin (DC-SIGN), involved in the capture and internalization of pathogens [83]. HSV-1 EVs carry the viral glycoprotein B that reduces the surface expression of HLA-DR, a MHC class II cell surface receptor, by directing it into the vesicular network to avoid viral recognition by the immune system [84]. Additionally, they transport different viral mRNAs and miRNAs [85].

### 4.1. The Case of HIV

Human immunodeficiency virus (HIV) is a retrovirus recognized as the etiological agent of Acquired Immune Deficiency Syndrome (AIDS), a progressive pathology that induces a weakening of the host immune system. The virus is characterized by two identical copies of a positive-sense single-stranded-RNA enclosed in a viral nucleocapsid, called a core, which is surrounded by a membrane envelope [86]. The genome codifies for three structural protein precursors, Gag, Pol and Env, the two regulatory proteins Tat and Rev, and the four accessory proteins Nef, Vif, Vpr, and Vpu. All these factors differently contribute to the establishment of HIV infection [87]. The main targets of the virus are the immune cells such as T helper lymphocytes, macrophages, microglial and dendritic cells, which express on their plasma membrane the CD4 receptor used by the virus to bind and enter the cells. HIV persists inside the host, leading to a progressive impairment of the immune system because of the depletion of CD4^+^ T helper cells, finally resulting in AIDS [86].

**Figure 2:**
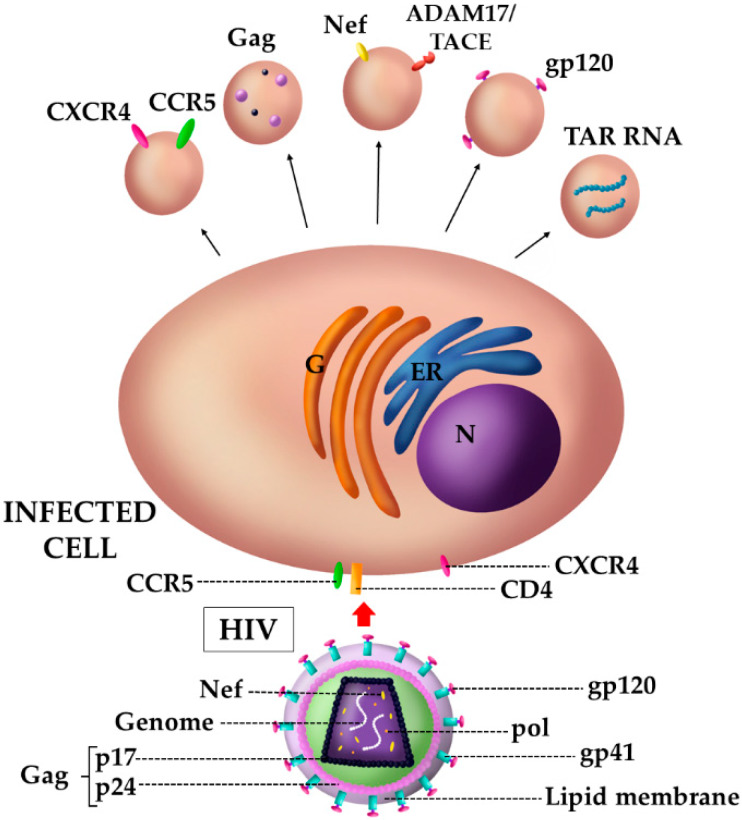
Schematic representation of EVs released by HIV-infected cells. EVs derived from HIV-infected cells carry both viral and host cell components that favor viral spreading and HIV-associated immune pathogenesis. Regarding host proteins, EVs transfer the two co-receptors CCR5 and CXCR4, used by the virus to mediate its entry, to null cells. Among viral components, EVs carry the proteins Gag, gp120 and Nef, as well as Trans-Activation Response (TAR) RNA, a pre-microRNA that produces mature microRNAs. Nef protein also induces the uploading into EVs of ADAM17/TACE, a TNFα converting enzyme. Nucleus (N); endoplasmic reticulum (ER); Golgi complex (G).

In recent years, different studies have highlighted the potential roles of EVs in HIV pathogenesis. The virus can take advantage of the endomembrane system not only by enhancing the viral biogenesis itself, but also by inducing EV biogenesis changes. These modifications may involve alterations in cargo composition, the frequency of EV release and targets, thus promoting viral spread, replication, and immune evasion (see Figure 2). In this respect, different studies have showed how EVs released from infected cells can deliver the HIV co-receptors CCR5 and CXCR4 to other cells, making them susceptible to the viral infection, probably increasing the tropism of the virus in vivo and favoring its dissemination [88,89]. Furthermore, EVs can facilitate infection through association with viral progeny, thus camouflaging the viral particles and reducing recognition by the immune system.

Regarding HIV–EV cargo, different viral proteins have been reported to be encapsulated in extracellular vesicles, including the virulence factor Nef, which has gained a great attention. Nef acts as molecular adaptor in both infected and uninfected cells by exerting multiple effects depending on its intracellular localization. The most important and best characterized functions of Nef are: (a) modulation of the expression of different surface receptors; (b) induction of a pre-activation state in CD4^+^ T cells, resulting in the increase in their susceptibility to the infection and promotion of viral gene expression; (c) regulation of apoptosis by inducing it in bystander uninfected cells meanwhile protecting infected cells; (d) regulation of the cytokine network contributing to chronic inflammation and, finally, (e) the increase in the infectivity of released HIV virions by preventing the incorporation of two antiviral proteins, SERINC3 and SERINC5 [87,90,91]. Interestingly, Nef also plays an important role in the vesicular network; it can influence the endosomal trafficking, being incorporated into MVBs, and induce late endosome formation. Not by chance, Nef binds and activates the PI3 kinase involved in vesicular formation [92]. In particular, Nef influences the production of vesicles and exploits them for its transport [93]. Different studies have shown how Nef increases vesicular production [94,95] and its association with EVs, which was observed both in in vitro and in vivo studies [94,95,96]. Interestingly, vesicles containing Nef turned out to exert multiple pathogenic effects: the induction of T-cell apoptosis [94]; the down-modulation of cell surface molecules (i.e., MHC-I and CD4) to favor immune evasion [97], and the restoration of the infectivity of HIV particles defective in Nef protein [98]. Furthermore, Nef binds and incorporates into vesicles the TNFα converting enzyme (ADAM17 or TACE) [99,100], a metalloprotease that cuts the pro-TNFα present in cell membranes, causing the release of the active form of TNFα. Nef–EVs, by inducing TNFα release, promote the activation of resting cells, such as CD4^+^ T lymphocytes, making them competent for HIV expression and replication [101,102,103]. A similar mechanism was also found to be involved in the reactivation of cells latently infected with HIV-1 [104]. These mechanisms have probably a great relevance in vivo, since Nef–EVs charged with ADAM17 and other pro-inflammatory factors appear to correlate with HIV-associated immune pathogenesis in both viremic and non-viremic chronic infection [99,103]. Noteworthy, HIV infection can also cause chronic neurological diseases and neurocognitive disorders (HIV-1 associated neurocognitive disorders (HAND)). Nef-containing EVs seem to be involved in the progression of these neuroimmune diseases. In chronic neurological diseases related to HIV infection, Nef–EVs released by infected microglia can disrupt the integrity of the blood–brain barrier, thus increasing its permeability, and can enhance the levels of some cytokines and chemokines such as IL-2, IL-8, IL-6, RANTES and IL-17A [105]. EVs isolated from the plasma of HAND patients can transport Nef protein and its correspondent mRNA is also able to induce the expression of the viral protein in a neuroblastoma cell line. This expression increases the production and secretion of beta amyloid protein, probably contributing to the cognitive impairment of HAND patients [96]. Overall, these data suggest that Nef–EVs are key mediators of the neuroimmune pathogenesis of HIV infection. Among the viral components transported into EVs, there is also Gag protein, which was found inside vesicles collected from infected Jurkat T cells, but its effects in uninfected cells are currently unknown [106]. Even gp120 envelope protein was found in EVs isolated from infected cells that seem to significantly increase the viral infectivity in human lymphoid tissues [107].

EVs released from HIV-infected cells can transport viral RNAs, which stimulate Toll like receptor-8 (TLR8) signaling to promote TNFα release, which may contribute to chronic immune activation [108]. Furthermore, the HIV Trans-Activation Response (TAR) RNA was found to be incorporated into EVs. TAR RNA is a microRNA precursor that matures after cleaving and its products are involved in apoptosis regulation and viral replication in infected cells. Once transferred to recipient cells, TAR microRNAs enhance the downregulation of pro-apoptotic proteins, thus supporting infected cells survival [109]. TAR–EVs have also been reported to modulate the gene expression of different pro-inflammatory cytokines, such as IL-6 and TNF-β, in human macrophages. These cytokines allow the maintenance of a continuous state of activation of target cells, probably favoring the efficient entry and replication of the virus [110]. In conclusion, EVs from different cell sources seem to play different roles in HIV pathogenesis. The effect of EVs depends on the cargo, the type of cell from which they originate, and their interaction with viral components.

### 4.2. The Case of HCV

**Figure 3:**
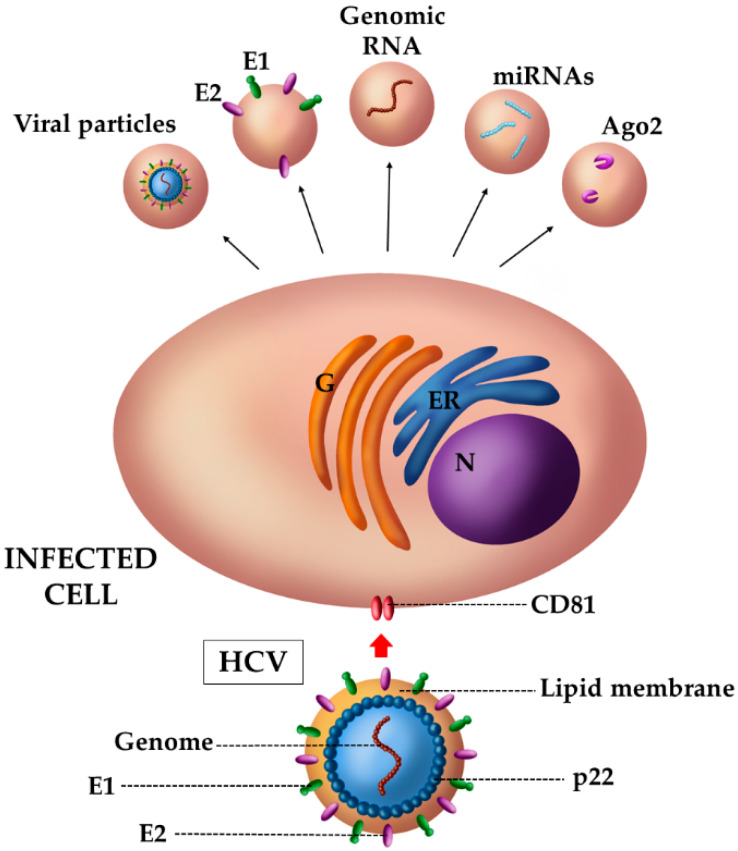
Schematic representation of EVs released by HCV-infected cells. EVs derived from HCV-infected cells carry both viral and host cell components that promote viral dissemination and immune pathogenesis. HCV-EVs carry entire viral particles, thus hiding them to immune system and allowing the virus to enter the recipient cells using uncanonical receptors. In addition, HCV-EVs transfer the glycoproteins E1 and E2, viral genomes as well as miRNAs, such as miRNA-19a, miRNA-192 and miRNA-122, and host proteins including Argonaute 2 (Ago2), the effector of RNA interference (RNAi) that associates with Hsp90. Nucleus (N); endoplasmic reticulum (ER); Golgi complex (G).

Hepatitis C Virus (HCV) is a human virus belonging to *Flaviviridae*, characterized by a positive single-stranded RNA of about 9.6 kb. The HCV genome codifies a precursor protein that is cleaved in ten viral proteins, including the core protein p22, the two glycoproteins of the viral envelope (i.e., E1 and E2) and the non-structural ones. This virus has hepatic tropism and it represents one of the main causes of liver damage, since it provokes chronic hepatitis in about 80% of infected people. The pathogenesis is mainly caused by an alteration of cytokines, chemokines, and growth factors, which favor the production of the extracellular matrix (ECM) and decrease its degradation by means of metalloproteases (MMPs). These events lead to liver fibrosis that, in a variable percentage of cases, can evolve into cirrhosis and in hepatocellular carcinoma (HCC) [111]. The study of the role of EVs during HCV infection is a field still in active growth. Despite this, some potential mechanisms of these vesicles have already been identified (see Figure 3). As previously mentioned, a peculiarity of EVs is their ability to transfer the virus, or parts of it, to other “naive” cells, thus becoming a vehicle of viral transmission [112]. It is known that viral progeny is usually composed by infective and defective viral particles. The latter, because of random mutations, may not necessarily lead to a productive infection. In these respects, the vesicular transport can represent a real advantage for the virus, because EVs can compensate for some shortcomings [113]. For instance, when viral particles defective in anchoring glycoproteins were carried inside EVs, they could enter target cells by means of cellular proteins present on EV membranes. In this way, EVs would allow the establishment of a productive infection for defective particles. Furthermore, different studies reported that HCV exploits the cellular vesicular pathway for the assembly and release of viral particles [114], and HCV-infected cells release vesicles containing E1 and E2 envelope proteins [115], the entire viral genome [116], or even entire viral particles [117]. These vesicles, once they enter target cells, can establish a productive infection exactly as with free viral particles [118]. Considering these data, we can imagine that EVs could represent an interesting and important advantage, from an evolutionary point of view, in the generation of viral “quasispecies”. The latter are collections of closely related viral genomes generated upon replication of RNA viruses, including HCV, and subjected to a continuous process of genetic variation and competition among the variants generated. Only the variants that fit best in a given environment are selected [113]. In this context, the EV cargo could help to establish a productive infection for those genomic variants that, otherwise, would be negatively selected due to the accumulated mutations that are incompatible with a successful infection. In this way, EVs might favor the survival of a major number of viral particles.

Therefore, as already reported above for HIV, this uncanonical communication pathway would represent, for the virus, an advantage to disguise itself and not be recognized by the immune system, and also to enter the recipient cells using receptors other than canonical ones [112].

However, during an HCV infection the EV cargo is not characterized exclusively by viral components. Indeed, cytokines and additional factors, which promote an efficient viral replication, have also been detected in EVs. One of these factors is represented by microRNAs (miRNAs), 21–23 nucleotide non-coding RNA molecules that post-transcriptionally repress gene expression [119]. Various types of miRNAs have been associated with liver tissue and some of them are highly specific. For example, EVs can transport and release miRNA-192, causing phenotypic changes in the cells because of the increased production of Transforming Growth Factor β (TGF-β). This cytokine is involved in the liver tissue maintenance of fibrogenesis and acts as a powerful activator of hepatic stellate cells (HSCs). TGF-β exists in three isoforms: TGF-β1, -2, -3, but the most representative of the fibrogenic pathways in the liver is TGF-β1 [120]. The latter usually reduces the degradation of the extracellular matrix (ECM) thanks to the production of MMP inhibitors (TIMPs), which block the activity of matrix metalloproteases (MMPs). However, the altered production of TGF-β leads to an increased transcription of pro-fibrotic molecules, such as alpha-smooth muscle actin (α-SMA) and collagen type I (COL1), through pathways involving MAPKs, JNK and Akt. These events finally cause the excessive formation of the extracellular matrix (ECM), which is responsible for the development of fibrous tissue [120]. A similar effect was observed following the release of miRNA-19a by EVs. HCV-EVs carry miR-19a and target SOCS3 in HSCs, which, in turn, activates the STAT3-mediated TGF-β signaling pathway and enhances fibrosis marker genes [111]. Indeed, non-canonical STAT3 activation induces a higher TGF-β1 and collagen I expression.

Furthermore, other factors such as Ago2, miR-122 and HSP90, have been found to intensify HCV replication [112]. miR-122 is one of the most abundant miRNAs in the liver tissue, representing about 70% of the total miRNA pool [119]. The key role of microRNAs is to regulate the translation of cellular mRNAs via their integration into a protein complex called RISC (RNA-induced silencing complex) with important proteins called Argonautes (Ago), of which human cells have four types: Ago1, -2, -3 and -4 [121]. Argonaute 2 (Ago2), the effector of RNA interference (RNAi), requires and associates with heat shock protein 90 (Hsp90). The latter is one of the heat shock proteins (Hsps), molecular chaperones that control the folding and function of proteins. However, it is necessary to underline that these factors potentiate viral replication, but they are not essential, since their inhibition does not prevent it.

In conclusion, these data demonstrate how the EVs released during HCV infection provide a variety of molecules that favor efficient viral replication in recipient cells [112,122].

### 4.3. The Case of SARS Viruses

Coronaviruses (CoVs) belong to a large family of enveloped RNA viruses involved in various respiratory syndromes. The name coronavirus derives from their characteristic electron microscopy appearance. They have a typical round “fringe” that recalls the solar corona, which surrounds a spherical enveloped particle containing the positive single-stranded RNA genome. The latter is complexed with the N viral protein, thus forming a helical symmetrical nucleocapsid complex [123]. These viruses have the largest known viral RNA genome (around 30 kb). All CoVs are characterized by a common set of structural proteins: the nucleocapsid (N), the spike (S), the membrane (M) and the envelope (E) proteins [124,125]. This type of viruses was known to cause mild to moderate diseases in humans, often characterized by cold-like symptoms and more rarely by the development of severe respiratory syndromes. However, some previously unknown species have caused epidemics with severe clinical conditions in the new millennium. This is the case of the severe acute respiratory syndrome virus (SARS-CoV), which emerged in southern China at the end of 2002; the Middle East respiratory syndrome virus (MERS-CoV), which emerged in Saudi Arabia in 2012; and now the severe acute respiratory syndrome coronavirus 2 (SARS-CoV-2), which originated in the city of Wuhan in China in December 2019 [126].

The interaction of the CoV S glycoprotein with its surface receptor is essential to determine the cellular host tropism. MERS-CoV S protein binds the human receptor dipeptidyl peptidase-4 (DPP4) or adenosine deaminase complexing protein 2, which is expressed on the surface of the cells of the airway system. A work carried out during the first SARS-CoV epidemic identified the human host factor angiotensin-converting enzyme 2 (ACE2) as the receptor for SARS-CoV [127]. ACE2 is a metalloprotease expressed in the epithelial and alveolar cells of the human lung, in the intestine, liver, heart, vascular endothelium and kidneys [128,129,130,131]. SARS-CoV-2 spike (S) protein has been experimentally shown to bind ACE2 in host cells with significantly higher affinity than SARS-CoV S [132,133]. The main host protease, which mediates S protein activation in primary target cells and allows viral entry, is the Type II transmembrane serine protease (TMPRSS2) [132,134,135,136,137,138]. Other host proteases, such as furin, have also been suggested to promote the pathogenesis of this pandemic SARS-CoV-2 clade, but when and where they process S protein remains to be determined [138,139,140,141]. ACE2 has a protective effect on lung safeguarding from acute injury in mouse models. The binding with SARS-CoV S induces a downregulation of ACE2 surface expression, and this mechanism possibly contributes to the severe pathogenesis of SARS viruses [142]. The immune response against CoVs has an important impact on the development of the pathogenesis. Indeed, in severe cases of SARS, the pathology is correlated to the hyperactivation of innate immune signaling. This event occurs in the abnormal production of interferons and high levels of pro-inflammatory cytokines such as IL-1, IL-6, IL-8, CXCL-10 and TNFα, which contribute to the lung damage. In patients with severe SARS disease, aberrant IFN, Interferon Stimulated Genes (ISGs), and cytokine responses were observed compared to healthy individuals, thus providing evidence that SARS is an innate immune response-regulated disease [143]. Recently, Ziegler et al. [144] have described that ACE2 is an ISG in specific human, but not murine, airway epithelial cells. Indeed, by treating primary human upper airway basal cells with distinct types of inflammatory cytokines, they unexpectedly observed that IFN-α drives the *ACE2* expression. This discovery, along with SARS-CoV-2 utilizing host ACE2 to gain entry to cells, suggests that SARS-CoV and SARS-CoV-2 may exploit the ACE2-mediated tissue-protective response to provide further cellular targets [144]. This potential strategy employed by SARS-CoV-2 could represent a unique challenge for the human host, as well as for HCoV-OC43, which targets the two restriction factors IFITM2 and IFITM3 [145]. Ziegler at al. provide a motivation to understand the specific role and balance of Type I and Type II IFNs, as well as Type III IFNs, in tissue protection during SARS-CoV-2 infection [144]. Carefully controlled clinical trials will be essential to determine the overall effects of different IFNs [146]. One study on SARS-CoV suggested the timing of the Type I IFN response as a key component of the in vivo response [147]. Clinical therapy using approved IFNs has been attempted for SARS-CoV, MERS-CoV and SARS-CoV-2 in the absence of a controlled trial and showed a mixed response, i.e., suggesting either rapid improvement or the worsening of symptoms [148,149,150].

After binding to cell surface receptors, CoVs enter cells through receptor-mediated endocytosis. Subsequently, the translation occurs and two large polyproteins are produced and cleaved. Different nonstructural proteins (Nsps), with enzymatic activity that are involved in the genome replication and expression, are generated. The Nsps are recruited together with host cell proteins to form the membrane-associated replication and transcription complexes (RTCs). The presence of RTCs allows the accumulation of different mRNAs that are translated in structural and non-structural viral proteins. Once they are produced in sufficient amounts, the virus is assembled and buds on the membrane of the endoplasmic reticulum (ER) and Golgi, where the membrane M, E and S viral proteins are accumulated. Finally, viral particles are released into the lumen of host cell membranous compartments and, afterwards, virions are released into the extracellular space via secretory pathways [131,151].

**Figure 4:**
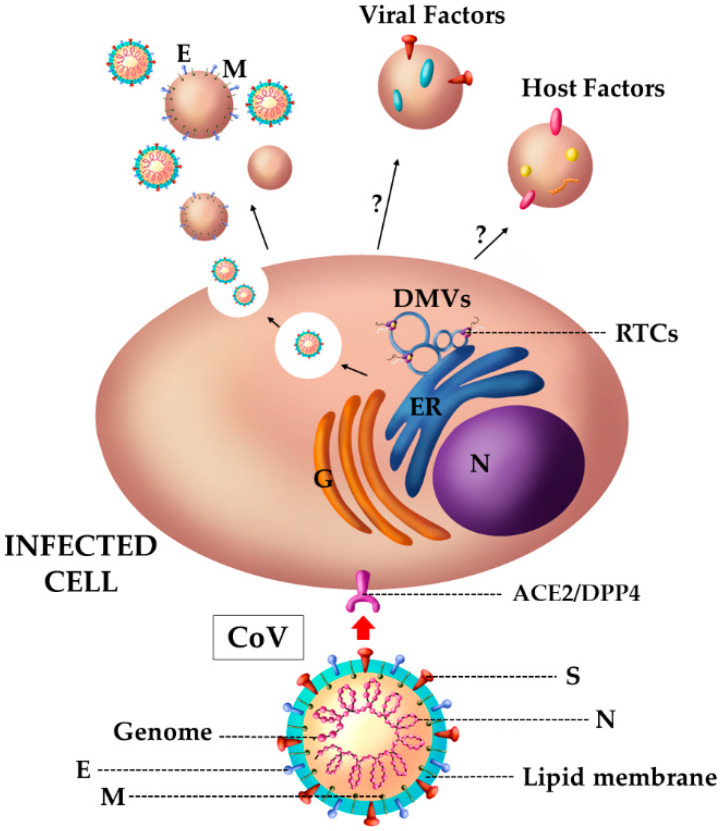
Schematic representation of EVs released by coronavirus (CoV)-infected cells. CoVs hijack the cellular machinery to favor their replication. CoVs proteins promote the formation into the cytosol of double-membrane vesicles (DMVs) that are associated to the replication and transcription complexes (RTCs) where the viral replication occurs. After the production of structural and non-structural proteins, the budding can take place from Golgi and ER membranes. Subsequently, viral particles are released into the extracellular space by exploiting the vesicular network. In addition to viral particles, CoVs induce the release of vesicles carrying the viral envelope (E) and membrane (M) proteins. To date, there are not clear evidences of vesicles released by CoV-infected cells transporting other viral or host factors. Nucleus (N); endoplasmic reticulum (ER); Golgi complex (G).

Although these second-millennium CoVs are some of the most pathogenically virulent human viruses in the world and a lot of research has been conducted on the first two, they are relatively new and thus there are several unanswered questions. For instance, the relationship between CoVs and EVs is still unclear and barely explored. In this respect, studies carried out on viral proteins and replicative strategies of these viruses suggest that CoVs hijack the vesicular release pathway in some way. It is possible to speculate that CoVs could influence EV release and composition (see Figure 4). Several research groups reported that coronavirus replication is strictly linked to intracellular vesicle formation, and the replicative complex binds the intracellular membrane, leading to the formation of vesicular structures. Two different vesicular structures have been identified: the first one corresponds to single-membrane spherules that are formed in membranous organelles, such as ER, peroxisomes or endosomes [152]; the second ones are double-membrane vesicles (DMVs) with a diameter of about 200–300 nm, which are often associated to other structures, such as tubules or ER membranes, thus forming a vesicular network in the cytosol [153,154,155,156,157,158]. The generation process of these structures is still not fully understood. Some research groups suggested that DMV formation could be correlated with the viral hijacking of the host’s autophagy machinery [159,160]. However, it is a common idea that different viral Nsps, thanks to their transmembrane domains and the fact that they are anchored to the membrane, can promote the formation of these structures. Interestingly, Nsp3, Nsp4 and Nsp6 SARS proteins are able to induce the formation of bilayer membrane vesicles in tissue cultures. Indeed, both the exogenous treatment with Nsp3 protein and the endogenous expression of Nsp3, Nsp4 and Nsp6 proteins may perturb the membrane network [161,162]. Moreover, the co-transfection of constructs for the expression of the three Nsps prompts the budding of vesicles in target cells. The phenotype obtained was very similar to the one observed during viral infection [161].

Other CoV proteins are involved in membrane morphological modifications. For instance, the S2 subunit of the spike glycoprotein, which is involved in the cellular attachment, possesses various membranotropic segments that induce membrane perturbation and could allow membrane negative curvature [163]. Additionally, it was reported that M and E glycoproteins can promote, by themselves, the formation and release of 100 nm “vesicles”, morphologically indistinguishable from viral particles. These data confirm the possibility of the production of nucleocapsidless particles during CoV infection [164]. As reported for other viruses, the production of vesicles together with the viral progeny could be a useful strategy to mask viral particles and transport viral factors to uninfected cells. In conclusion, these observations suggest that CoVs, like other viruses, exploit the cellular pathways to produce EVs, even if, to date, there is no clear evidence of their induction during CoV infection in vivo.

## 5. New Therapeutic and Vaccination Strategies Using Extracellular Vesicles

EVs are not only vehicles that can promote viral progression and pathogenesis, but are also important immunostimulatory structures acting as mediators of immune responses. In this regard, it was found that vesicles from dendritic cells (DCs), carrying the major histocompatibility complexes MHC-I and -II, as well as costimulatory molecules such as CD80 and CD86, can induce CD8^+^ and CD4^+^ T lymphocyte activation [165]. Different groups have explored how EVs modulate the immune system in various pathological conditions [34,166]. In the context of infections, for instance, the transport of viral components can make EVs a double-edged sword: on the one hand, they support viral spreading and pathogenesis, while, on the other hand, they can potentially transfer viral antigens to immune cells and be responsible for the induction of adaptive immunity that is able to counteract the viral spreading [167].

Furthermore, EVs released from infected cells can be charged with cellular proteins that have potent antiviral activities. This is the case of APOBEC3G, a cytidine deaminase that has an important role in restricting HIV replication. In infected cells, APOBEC3G is counteracted by the expression of the viral accessory protein Vif. The latter mediates the polyubiquitination and rapid proteasomal degradation of ABOBEC3G, thus preventing its incorporation into the progeny virus nucleocapsid. Uninfected cells can transport APOBEC3G through EVs to infected cells, in which it deaminates deoxycytidines in the minus-DNA strand that is formed during reverse transcription. This results in a high rate of nucleotide base substitutions (G-to-A transition) or the premature termination of reverse transcription that is incompatible with viral viability [168]. Additionally, vesicles released by HSV-1-infected cells transport molecules of the innate immune system such as the stimulator of interferon genes (STING), which establishes an antiviral response in target cells [169].

The growing body of evidence indicating the capability of EVs to promote an immune response increased interest in the use of vesicles as potential therapeutic or diagnostic tools. In this regard, EVs are considered excellent biomarker candidates that hold great potential for the detection of many pathological conditions, due to their ability to alter their cargo according to different cell stimuli. Moreover, since EVs are present in many biological fluids, they are easily accessible for liquid biopsy [170]. Another aspect that has gained considerable interest in the scientific community is the potential use of EVs as drug delivery vehicles. In fact, EVs offer distinct advantages as gene therapy delivery vectors since they possess cellular membranes with multiple adhesive proteins on their surface. Their small size and flexibility enable them to cross major biological barriers, such as the blood–brain barrier. Their potential utility in drug delivery is also due to their intrinsic homing capacity. Unlike liposome formulations and lentiviral-based delivery systems, EVs are naturally secreted by cells and thus they possess a high biocompatibility, safety and stability in circulation, which allow them to overcome many of the limitations of cell-based therapeutics. In this regard, it was demonstrated that EVs are able to deliver the anti-inflammatory agent curcumin that, in this form, was found to be more stable than free curcumin [171].

To date, some studies have used EVs-based therapeutics to treat diseases by engineering EVs with full-length proteins that were effective in inducing specific, unrestricted cytotoxic T cell (CTL) immunity when injected in mice. Specifically, they use a DNA vector for the expression of HIV-1 Nefmut peptide fused with human papillomavirus E7 viral antigen. Nefmut peptide allows the anchoring of the antigen to EV membranes and increases the production of vesicles in target cells. The intramuscular injection of the vector prompts the spontaneous production of EVs charged with the foreign protein fused to Nefmut, which appears to induce an important immunological response [172]. Subsequently, the same group explored the possibility to use this vesicle-based vaccine platform with different viral antigens such as Ebola virus VP24, VP40 and NP, Influenza virus NP, HCV NS3 and others. All the antigens tested were detected in engineered EVs that triggered the expected immunological response [173]. Another research group has proposed the engineering of the SARS-CoV S protein with VSV-G protein to generate an expressing vector for a chimeric receptor protein, because the S wild type protein of SARS was not detected in vesicles isolated from transfected HEK293 cells. Through transfection procedures, they obtained a cell population expressing the fusion protein and producing EVs charged with the S chimeric protein. When engineered EVs were injected in mice, the induction of high levels of neutralizing antibodies was observed [174].

In conclusion, EVs appear to be promising bio-nanoparticles for the development of new therapeutic, diagnostic and prophylaxis strategies against microbial infections and other pathologies. This emerging field has a good chance to provide relevant results for the treatment of different viral infections.

## 6. Conclusions

EVs are important mediators of cell-to-cell communication both in physiological and pathological conditions. Their importance is due to EVs’ capacity to transport different biological molecules such as lipids, proteins, or nucleic acids to target cells, where they can promote a wide range of effects. Many studies have suggested that EVs and viruses are not so distant as one might imagine and, in some respects, they seem to be close relatives. During infections, many viruses take advantage of EVs by hijacking their vesicular biogenesis machinery; they modify EVs by incorporating specific viral factors that contribute to create a suitable environment for viral infection. HIV, HCV and SARS viruses are three representative examples of how viruses, in different ways, can exploit EV production to favor their survival and diffusion. As reported in this review, different structural proteins such as Gag and gp120 for HIV, the proteins E1 and E2 for HCV, and the proteins E and M for coronaviruses, have been detected inside EVs, probably as a consequence of their involvement in viral budding [106,107,115,164]. The generation of vesicles like viral particles during the infection could be a helpful strategy for the virus to avoid recognition by the immune system. Moreover, EVs can transport non-structural proteins and other viral factors, such as Nef [93,94,95,96,97,98] and/or the TAR RNA of HIV [109,110], or even the entire viral genome as in the case of HCV [116]. Both HIV and HCV are able to hijack the cellular system by redirecting cellular proteins and nucleic acids into EVs to create an appropriate environment for their own replication and spread. For example, HIV–EVs transport the viral co-receptors CCR5 and CXCR4 to other cells to extend the pool of target cells available for the virus [88,89], and the metalloprotease ADAM17, which is involved in the inflammatory response [99,100], whereas HCV-EVs are charged with Ago2 and miRNAs [111,112,119,120]. The delivery of cellular and viral proteins through EVs exerts multiple effects on target cells. HIV-TAR RNA, for example, produces microRNAs with anti-apoptotic effects, helping the survival of infected cells [110], whereas HIV-Nef EVs and HCV-Ago2-miRNA122 EVs act in target cells by enhancing viral replication. Moreover, HIV–EVs containing Nef, ADAM17 or TAR RNA and HCV-EVs with miRNA-192 and miRNA-19a have been associated to the perturbation of the cytokine/chemokine network [101,102,103,104,105,111,112,120]. Many of these effects allow the preservation of cell activation, thus favoring viral entry and replication, and often contributing to the progression of the pathogenesis. To date, few in-depth studies concerning vesicles and coronaviruses have been reported in the literature, since these viruses have only recently attracted the interest of the scientific community. Therefore, this field is certainly open to new studies and coronaviruses could significantly affect EV content. Finally, viral EVs not only help pathogens, but they are also recognized and used by the host to induce innate and adaptive immune responses to viral infections. Ongoing research will pave the way for therapeutic and/or prophylaxis strategies that exploit EVs.
